# Supplementary material for: Nobody ever questions—Polypharmacy in care homes: A mixed methods evaluation of a multidisciplinary medicines optimisation initiative
Source: PLoS One. 2021 Jan 7;16(1):e0244519. doi: 10.1371/journal.pone.0244519 (PMC7790299; doi:10.1371/journal.pone.0244519)
Supplement: S1 File — (DOCX) [file pone.0244519.s001.docx]

S1 File. Revised Standards for Quality Improvement Reporting Excellence 

SQUIRE 2.0

| Notes to Authors | |  |
| --- | --- | --- |
| - The SQUIRE guidelines provide a framework for reporting new knowledge about how to improve healthcare. - The SQUIRE guidelines are intended for reports that describe [system](http://www.squire-statement.org/index.cfm?fuseaction=page.viewpage&pageid=485#System) level work to improve the quality, safety, and value of healthcare, and used methods to establish that observed outcomes were due to the [intervention(s).](http://www.squire-statement.org/index.cfm?fuseaction=page.viewpage&pageid=485#Interventions) - A range of approaches exists for improving healthcare.  SQUIRE may be adapted for reporting any of these. - Authors should consider every SQUIRE item, but it may be inappropriate or unnecessary to include every SQUIRE element in a particular manuscript. - The SQUIRE Glossary contains definitions of many of the key words in SQUIRE. - The [Explanation and Elaboration](http://www.squire-statement.org/index.cfm?fuseaction=page.viewpage&pageid=504)document provides specific examples of well-written SQUIRE items, and an in-depth explanation of each item. - Please cite SQUIRE when it is used to write a manuscript. | |  |
| Title and Abstract | |  |
| **1.  Title** | Indicate that the manuscript concerns an [initiative](http://www.squire-statement.org/index.cfm?fuseaction=page.viewpage&pageid=485#Initiative) to improve healthcare (broadly defined to include the quality, safety, effectiveness, patient-centeredness, timeliness, cost, efficiency, and equity of healthcare)  **The title contains ‘Initiative’** |  |
| **2.  Abstract** | a.  Provide adequate information to aid in searching and indexing  b.  Summarize all key information from various sections of the text using the abstract format of the intended publication or a structured summary such as: background, local [problem](http://www.squire-statement.org/index.cfm?fuseaction=page.viewpage&pageid=485#Problem), methods, interventions, results, conclusions  we have used ‘implications’ rather than conclusions |  |
| Introduction | *Why did you start?* |  |
| [**3. Problem Description**](http://www.squire-statement.org/index.cfm?fuseaction=page.viewpage&pageid=485#Problem) | Nature and significance of the local [problem](http://www.squire-statement.org/index.cfm?fuseaction=page.viewpage&pageid=485#Problem)  **Page 3 Introduction** |  |
| **4. Available Knowledge** | Summary of what is currently known about the [problem](http://www.squire-statement.org/index.cfm?fuseaction=page.viewpage&pageid=485#Problem), including relevant previous studies  **Page 3 Background** |  |
| **5. Rationale** | Informal or formal frameworks, models, concepts, and/or [theories](http://www.squire-statement.org/index.cfm?fuseaction=page.viewpage&pageid=485#Theory) used to explain the [problem](http://www.squire-statement.org/index.cfm?fuseaction=page.viewpage&pageid=485#Problem), any reasons or [assumptions](http://squire.citysoft.org/index.cfm?fuseaction=page.viewPage&pageID=485&nodeID=1#assumptions) that were used to develop the [intervention(s),](http://www.squire-statement.org/index.cfm?fuseaction=page.viewpage&pageid=485#Interventions)and reasons why the [intervention(s)](http://www.squire-statement.org/index.cfm?fuseaction=page.viewpage&pageid=485#Interventions) was expected to work  **Page 3**  **Why the intervention is expected to work: lines 110-114 references 2-7**  **Why this intervention chosen : lines 80-83** |  |
| **6. Specific Aims** | Purpose of the project and of this report  **Page 5 ‘Aims’** |  |
| Methods | *What did you do?* |  |
| [**7. Context**](http://www.squire-statement.org/index.cfm?fuseaction=page.viewpage&pageid=485#context) | Contextual elements considered important at the outset of introducing the [intervention(s)](http://www.squire-statement.org/index.cfm?fuseaction=page.viewpage&pageid=485#Interventions)  **Setting - P 6, Approach & Recruitment**  **Physical environment or Setting – P5 ‘Setting’**  **Sense – making/interpretation of the environment and intervention: Setting p.5, Recruitment & retention p.9, Participants p.9.** |  |
| [**8. Intervention(s)**](http://www.squire-statement.org/index.cfm?fuseaction=page.viewpage&pageid=485#Interventions) | a.  Description of the [intervention(s)](http://www.squire-statement.org/index.cfm?fuseaction=page.viewpage&pageid=485#Interventions) in sufficient detail that others could reproduce it **P 6** Our intervention: ADRe-p **Fig 1 ‘How ADRe- works’ Fig 3a, 3b ‘Problems and solutions’** |  |
| **9. Study of the Intervention(s)** | **a**.  Approach chosen for assessing the impact of the [intervention(s)](http://www.squire-statement.org/index.cfm?fuseaction=page.viewpage&pageid=485#Interventions)  **P5 ‘Study Design’**  **b.  Approach used to establish whether the observed outcomes were due to the**[**intervention(s)**](http://www.squire-statement.org/index.cfm?fuseaction=page.viewpage&pageid=485#Interventions)  **p7 ‘Data Collection’, but also discussed under ‘limitations’ p.23**  **Tables 4 and S2 file, table D** |  |
| **10. Measures** | a.  Measures chosen for studying [processes](http://www.squire-statement.org/index.cfm?fuseaction=page.viewpage&pageid=485#Process) and outcomes of the [intervention(s),](http://www.squire-statement.org/index.cfm?fuseaction=page.viewpage&pageid=485#Interventions)including rationale for choosing them, their operational definitions, and their validity and reliability  b.  Description of the approach to the ongoing assessment of contextual elements that contributed to the success, failure, efficiency, and cost  c.  Methods employed for assessing completeness and accuracy of data  **a. page 7 ‘outcome measures’**  **b and c. p.7 ‘Data Collection’**  **c. see references 2 & 7 for further discussion** |  |
| **11. Analysis** | a.  Qualitative and quantitative methods used to draw [inferences](http://www.squire-statement.org/index.cfm?fuseaction=page.viewpage&pageid=485#Inferences) from the data, Methods for understanding variation within the data  **p.8 ‘Analysis ‘**  **Description of clinical findings para 1, interviews para 2** |  |
| **12. Ethical Considerations** | [Ethical aspects](http://www.squire-statement.org/index.cfm?fuseaction=page.viewpage&pageid=485#Ethical_aspects)of implementing and studying the [intervention(s)](http://www.squire-statement.org/index.cfm?fuseaction=page.viewpage&pageid=485#Interventions) and how they were addressed, including, but not limited to, formal ethics review and potential conflict(s) of interest  **P8 ‘Ethics’** |  |
| Results | *What did you find?* |  |
| **13. Results** | a.  Initial steps of the [intervention(s)](http://www.squire-statement.org/index.cfm?fuseaction=page.viewpage&pageid=485#Interventions) and their evolution over time (e.g., time-line diagram, flow chart, or table), including modifications made to the intervention during the project  p.6 ‘Developing ADRe-p’, S2 file, Table A  **P 7 ‘Approach and Recruitment’ p.9 Recruitment & retention, Fig 2a, 2b**  b.  Details of the [process](http://www.squire-statement.org/index.cfm?fuseaction=page.viewpage&pageid=485#Process) measures and outcome  **P. 10 ‘outcomes’ Tables 1-3. All changes are listed in Table 3. S2 file, Tables B-D.**  **Clinical Impact listed Table 3 all 19 participants**  c.  Contextual elements that interacted with the [intervention(s)](http://www.squire-statement.org/index.cfm?fuseaction=page.viewpage&pageid=485#Interventions)  **‘Making it happen’ theme 3 p.18**  d.  Observed associations between outcomes, interventions, and relevant contextual elements   **Tables 2-4, S2 file Table D, Fig 2a**  e.  Unintended consequences such as unexpected benefits, [problems](http://www.squire-statement.org/index.cfm?fuseaction=page.viewpage&pageid=485#Problem), failures, or costs associated with the [intervention(s).](http://www.squire-statement.org/index.cfm?fuseaction=page.viewpage&pageid=485#Interventions)  **p.20** The economic costs and benefits of ADRe-p.  **Unsolved problems, Table 4, Fig 2 b, Table D**  f.  Details about missing data  **Table 2, S2 file, tables B, C** |  |
| Discussion | *What does it mean?* |  |
| **14. Summary** | a.  Key findings, including relevance to the [rationale](http://www.squire-statement.org/index.cfm?fuseaction=page.viewpage&pageid=485#Rationale) and specific aims  **p.21 Discussion, para 1. Key findings - ‘Discussion’, linking to rationale; linking to aims ‘Implications’ p.24**  b.  Particular strengths and weaknesses of the project  **‘Limitations and Strenghts’ p.23** |  |
| **15. Interpretation** | 1. Nature of the association between the [intervention(s)](http://www.squire-statement.org/index.cfm?fuseaction=page.viewpage&pageid=485#Interventions) and the outcomes   **Table 3, table D fig 2a, 2b Discussion of causality ‘Limitations’ lines 535 et seq**   1. Comparison of results with findings from other publications   **lines 472 et seq**   1. Costs and strategic trade-offs, including [opportunity costs](http://www.squire-statement.org/index.cfm?fuseaction=page.viewpage&pageid=485#Opportunity_costs)   **‘A difficult and orphaned task’ p.22**  **Resource use p.20 Economic Costs & lines 562 et seq** |  |
| **16. Limitations** | a.  Limits to the [generalizability](http://www.squire-statement.org/index.cfm?fuseaction=page.viewpage&pageid=485#Generalizability) of the work  **‘Limitations’ line 548 et seq**  b.  Factors that might have limited [internal validity](http://www.squire-statement.org/index.cfm?fuseaction=page.viewpage&pageid=485#Internal_validity) such as confounding, bias, or imprecision in the design, methods, measurement, or analysis  **Discussion of volunteer bias, aetiology of adverse events – ‘Limitations’ line 558 et seq, line 540 et seq**  c.  Efforts made to minimize and adjust for limitations  **limitations lines 540 et seq** |  |
| **17. Conclusions** | 1. Usefulness of the work   ‘New ideas in old structures’ p.23   1. Sustainability   **Lines 527 et seq**  c.  Potential for spread to other [contexts](http://www.squire-statement.org/index.cfm?fuseaction=page.viewpage&pageid=485#context)  **lines 517 et seq**   1. Implications for practice and for further study in the field -   **Implications p.24**  e.  Suggested next steps  **Implications p.24** |  |
| Other Information |  |  |
| **18. Funding** | Sources of funding that supported this work. Role, if any, of the funding organization in the design, implementation, interpretation, and reporting  Financial Statement |  |
