# Supplementary material for: Nobody ever questions—Polypharmacy in care homes: A mixed methods evaluation of a multidisciplinary medicines optimisation initiative
Source: PLoS One. 2021 Jan 7;16(1):e0244519. doi: 10.1371/journal.pone.0244519 (PMC7790299; doi:10.1371/journal.pone.0244519)
Supplement: S2 File. Supplementary tables: Table A. Changes made from ADRe to ADRe-p. Table B. ADRe-p Profile items and responses: Vital signs (n = 19). Table C. ADRe-p Profile items and responses: Observations and questions (n = 19). Table D. Medicines and reported problems in first and last profiles — (DOCX) [file pone.0244519.s002.docx]

## S2 File: Supplementary Tables

## Table A. Changes made from ADRe to ADRe-p

- Changed parameters for Postural Hypotension item from ‘>19mmHg’ to **‘≥20mmgHg’**
- Added **‘Dehydration from HR Standing difference’** item
- Changed parameters for Girth from ‘<88 or <102cms’ to **‘female ≤88 or ≥80/88, male ≤94 or ≥94/102 cms’**
- Changed parameters for Temp from ‘36.1-38’ to **‘36.4-37.2’**
- Added the **‘Lung function tests available’** item - new
- Moved **‘Cognitive Decline’** item to ‘Observations’ section underneath ‘Balance’
- Moved all **‘Skin Observations or questions’** items to underneath ‘Feeling the cold’ item
- Added **‘Broken skin/poor healing’** item to Skin Observations section, underneath ‘acne’ - new
- Added **‘Other skin abnormalities’** item - new
- Removed **‘Irritability’** item (irritability is part of Aggression item in ADRe-p) - lost
- Removed **‘Hyperactivity’** item (hyperactivity is part of Agitation/anxiety item in ADRe-p) - lost
- Added **‘Pain’** item below ‘headaches’
- Moved **‘Nonverbal pain indicators’** item to below ‘Pain’
- Rearranged the order of/to**: ‘Tingling’** and then **‘Tinnitus’**
- Added **‘Vision/eyesight problems’** and **‘Dry eyes’** items below ‘Tinnitus’
- Removed **‘Burning urine/UTI’** item as this comes under ‘GU tract’ on ADRe-p - lost
- Added **‘Urinary catheter’** item below ‘GU tract’ - new
- Added **‘Childbearing age’** and **Pre-conception care’** items below ‘Problems with reproductive system’
- Added ‘**High salt intake’** item below ‘Short of breath’ - new
- Moved **‘Dental problem’, ‘Dry Mouth’, ‘Halitosis’, ‘Swallowing Difficulties’, ‘Indigestion/heartburn’** items to GI tract section below ‘High salt intake’
- Added **‘Respiratory Problems’** item below ‘Sore Throat’ - new
- Re-ordered **‘Prevention and Health** promotion’ items to reflect order on ADRe-p
- Added **‘2 or more meals eaten daily’** item below ‘Intake: misses meals’
- Added **‘Immunisations up to date’** item below ‘Glasses for bright sunlight’ - new

7 New items

3 items removed & subsumed under other sections

## Table B. ADRe-p Profile items and responses: vital signs (n=19)

| **Item on ADRe-p 1^st^ profile** | **Problem** | | **Missing** | **Problem on ADRe-p final profile** | | **Missing** | **Comments/examples** |
| --- | --- | --- | --- | --- | --- | --- | --- |
|  | **No** | **Yes** |  | **No** | **Yes** |  |  |
| Heart rate problem recorded | 18 | 1 | 0 | 18 | 1 | 0 |  |
| HR within normal limits (50-90 bpm) | 1 | 18 | 0 | 1 | 18 | 0 | Sitting in wheelchair, lying in bed |
| Irregular heart rhythm | 12 | 0 | 7 | 11 | 0 | 8 | None |
| BP lying or sitting problem marked | 14 | 5 | 0 | 12 | 6 | 1 |  |
| BP within normal limits (SBP 111-140 mmHg) | 5 | 14 | 0 | 6 | 12 | 1 | 3 >141, 3<111 for 1^st^ profiles  1 >141, 5<111 for 2^nd^ profiles  Sitting in chair/wheelchair, lying in bed |
| BP standing problem marked | 2 | 0 | 17 | 4 | 0 | 15 |  |
| Postural hypotension (SBP difference ≥20mmgHg) | 2 | 0 | 17 | 4 | 0 | 15 | Many residents noted as unable to stand, 1 noted as being not compliant to stand |
| Dehydration (HR standing difference >10%) | 2 | 1 | 16 | 2 | 0 | 17 | Fully hoisted, unable to stand for a long time |
| Weight BMI within normal limits | 13 | 4 | 2 | 14 | 3 | 2 | Weighed weekly, weight gain/loss, stayed the same |
| Weight change | 0 | 6 | 13 | 0 | 12 | 7 | 3 loss, 3 gain (highest 2.35kg) for 1^st^ profiles  4 loss (highest -3.1kg), 8 gain for 2^nd^ profiles |
| Girth problem indicated | 0 | 3 | 16 | 0 | 3 | 16 |  |
| Girth within normal limits (female ≤88 or ≥80/88, male ≤94 or ≥94/102 cms) | 3 | 0 | 16 | 3 | 0 | 16 | Some residents refused/did not want to co-operate |
| Temperature problem | 9 | 8 | 2 | 11 | 8 | 0 |  |
| Temperature within normal limits (36.4-37.2) | 8 | 9 | 2 | 8 | 11 | 0 | 8 temps lower than 36.4 (lowest at 34.7) for 1^st^ profiles  8 temps lower than 36.4 (lowest at 35.2) for 2^nd^ profiles  No temps >37.2 |
| Oxygen saturation problem marked | 14 | 4 | 1 | 15 | 4 | 0 |  |
| Oxygen saturation within normal limits (>96%) | 4 | 14 | 1 | 4 | 15 | 0 | Lowest oxygen saturation 79 for 1^st^ profiles  Lowest oxygen saturation 93 for 2^nd^ profiles |
| ECG available | 15 | 0 | 4 | 16 | 0 | 3 | None |
| Lung function tests available | 13 | 2 | 4 | 16 | 0 | 3 | None |

## Table C. ADRe-p Profile items and responses: observations and questions (n=19)

| **Item on PADRe 1st profile** | **Problem 1^st^ profile** | | **Missing** | **Problem on PADRe 2^nd^ profile** | | | **Missing** | **Comments/examples** |  |  |  |
| --- | --- | --- | --- | --- | --- | --- | --- | --- | --- | --- | --- |
|  | **No** | **Yes** |  | **No** | **Yes** | |  |  |  |  |  |
| Hand tremor | 15 | 4 | 0 | 11 | 8 | | 0 | Stiffness, unable to assist self, shaking, parkinson’s & MS. |  |  |  |
| Tongue tremor | 19 | 0 | 0 | 18 | 1 | | 0 | Jumpy, grinds teeth |  |  |  |
| Feet shuffling | 14 | 3 | 2 | 13 | 6 | | 0 | Immobile, bed bound, dementia decline |  |  |  |
| Abnormal movement resting | 16 | 3 | 0 | 16 | 3 | | 0 | Restless, jumpy, observed in lower extremities, tremors in hands & legs |  |  |  |
| Posture abnormal | 14 | 5 | 0 | 12 | 6 | | 1 | Cervical spine, bed bound, wedges used in bed, slouches, leans to the left |  |  |  |
| Gait abnormal | 9 | 1 | 9 | 10 | 1 | | 8 | Unable to walk, immobile, bed bound, very small steps, full hoist for transfers |  |  |  |
| Balance | 11 | 8 | 0 | 8 | 7 | | 4 | Needs full assistance with ADL, is in a wheelchair, falls, dementia decline, bed bound, uses stand aid |  |  |  |
| Cognitive decline | 4 | 13 | 2 | 9 | 10 | | 0 | Repetition, post cardiac arrest, due to MS, short term memory loss, dementia |  |  |  |
| Feeling cold | 14 | 5 | 0 | 11 | 8 | | 0 | Elderly, very thin, always feels cold |  |  |  |
| Bleeding/bruising | 17 | 2 | 0 | 17 | 2 | | 0 | Falls, puncture wound sustained from last fall, on Edoxaban tablets |  |  |  |
| Rash | 17 | 2 | 0 | 18 | 1 | | 0 | Prone to cellulitis, Lupus, occasional |  |  |  |
| Swelling | 15 | 4 | 0 | 15 | 4 | | 0 | Gout, prone to cellulitis, Ankles, Pedal oedema, both feet, GP advises to wear stocking |  |  |  |
| Sweating | 19 | 0 | 0 | 17 | 2 | | 0 | At times when restless, window open and fan on |  |  |  |
| Acne | 19 | 0 | 0 | 19 | 0 | | 0 | None |  |  |  |
| Broken skin | 15 | 3 | 1 | 16 | 3 | | 0 | Leg ulcers, sustained from last fall, graze area on shin |  |  |  |
| Hair loss | 16 | 3 | 0 | 16 | 3 | | 0 | Is quite bald anyway |  |  |  |
| Other skin abnormalities | 18 | 1 | 0 | 17 | 2 | | 0 | Old age spots, Lupus red sore and painful |  |  |  |
| Injection site problems | 16 | 0 | 3 | 19 | 0 | | 0 | None |  |  |  |
| Convulsions | 16 | 3 | 0 | 19 | 0 | | 0 | Seizures recorded for 3 residents |  |  |  |
| Behavioural problems | 10 | 8 | 1 | 11 | 7 | | 1 | Sometimes unsettled, anxious and frustrated, restless, aggressive, linked with dementia, very vocal/verbal, increased confusion, agitation |  |  |  |
| Self-harm | 19 | 0 | 0 | 19 | 0 | | 0 | None |  |  |  |
| Physical violence | 16 | 3 | 0 | 16 | 3 | | 0 | None |  |  |  |
| Aggression | 12 | 6 | 1 | 15 | 4 | | 0 | None |  |  |  |
| Agitation | 9 | 10 | 0 | 9 | 10 | | 0 | At times/occasionally, nervous, gets anxious if dad is late coming, worries about dying, of the future (CA breast) |  |  |  |
| Restlessness | 15 | 3 | 1 | 17 | 2 | | 0 | Previously, occasionally |  |  |  |
| Panic attacks | 17 | 2 | 0 | 19 | 0 | | 0 | None |  |  |  |
| Confusion | 7 | 12 | 0 | 11 | 8 | | 0 | Dementia, confused due to MS, occasional |  |  |  |
| Mood fluctuations | 12 | 7 | 0 | 9 | 10 | | 0 | Due to MS/Dementia, more vocal in PM, worries for dad and says she cannot see, health, missing home/family, antidepressants, wants to see what’s happening outside |  |  |  |
| Low energy | 10 | 9 | 0 | 13 | 6 | | 0 | Very/quite sleepy, states always tired, when she feels sad |  |  |  |
| Hallucinations | 15 | 3 | 1 | 16 | 3 | | 0 | Dementia, visual, reassured (GP is aware) |  |  |  |
| Sleep problems | 12 | 7 | 0 | 16 | 3 | | 0 | Days into nights, sometimes, variable sleep pattern, can be restless at times |  |  |  |
| Sedation | 15 | 3 | 1 | 17 | 2 | | 0 | Not usually, sleeps excessively without sedatives, GP has been informed of excessive sleep |  |  |  |
| Dizziness | 17 | 2 | 0 | 17 | 2 | | 0 | Risk of falls marked as assessed for 3 residents |  |  |  |
| Falls | 15 | 4 | 0 | 17 | 2 | | 0 | Some residents falls noted as being documented in care plan or main file/record |  |  |  |
| Headaches | 18 | 1 | 0 | 16 | 3 | | 0 | Cannot vocalise pain, in back, at times/occasionally |  |  |  |
| Pain | 6 | 13 | 0 | 12 | 7 | | 0 | Prescribed medications noted: Paracetamol, Ibuprofen, Butrans, Pregabalin, MCGH, Codeine, Fenbid gel |  |  |  |
| Non-verbal pain indicators | 16 | 3 | 0 | 17 | 2 | | 0 | Crying, scratching, abbey pain scale assessed |  |  |  |
| Tingling | 19 | 0 | 0 | 19 | 0 | | 0 | None |  |  |  |
| Tinnitus/hearing problems | 17 | 2 | 0 | 18 | 1 | | 0 | Hearing aids |  |  |  |
| Vision/eyesight problems | 5 | 14 | 0 | 4 | 15 | | 0 | Blind in left eye, wears glasses, cortically blind post cardiac arrest, has exotropia in eyes, posture, cataract, wears bifocal spectacles, |  |  |  |
| Dry eyes | 18 | 1 | 0 | 18 | 1 | | 0 | Sticky eyes |  |  |  |
| Urination problem/incontinence | 9 | 10 | 0 | 12 | 7 | | 0 | Incontinent, doubly incontinent, suprapubic catheter, suffers with regular UTI, urinary retention |  |  |  |
| Urinary catheter | 15 | 3 | 1 | 16 | 3 | | 0 | 3 residents indicated to have long term suprapubic catheters |  |  |  |
| Problems with reproductive system | 15 | 1 | 3 | 17 | 2 | | 0 | CA breast |  |  |  |
| Childbearing age | 16 | 2 | 1 | 15 | 2 | | 2 | Not sexually active, n/a |  |  |  |
| Pre-conception care | 17 | 0 | 2 | 18 | 0 | | 1 | None |  |  |  |
| Chest pain | 19 | 0 | 0 | 19 | 0 | | 0 | None |  |  |  |
| Short of breath | 16 | 3 | 0 | 17 | | 2 | 0 | Sometimes when unsettled, at times (asthmatic), anxiety, nervous, occasionally after mobilising |  |  |  |
| High salt intake | 19 | 0 | 0 | 19 | | 0 | 0 | Of those with high salt intake, only 1 resident was noted as being short of breath on both 1^st^ and 2^nd^ profiles |  |  |  |
| Dental problem | 14 | 4 | 1 | 13 | | 6 | 0 | Painful teeth and gums, refused oral care, has dentures, dental cavities, not always co-operative with oral hygiene practices, poor oral health due to behaviour regular dental checks |  |  |  |
| Dry mouth | 17 | 2 | 0 | 18 | | 1 | 0 | Due to Sinemed |  |  |  |
| Halitosis | 18 | 1 | 0 | 18 | | 1 | 0 | None |  |  |  |
| Hyper-salivation | 17 | 2 | 0 | 17 | | 2 | 0 | It was noted for 3 residents that hyper salivation did not occur with respiratory tract infection  Some noted as drooling due to posture |  |  |  |
| Swallowing difficulties | 13 | 6 | 0 | 16 | | 3 | 0 | Only 1 assessment date noted for residents with swallowing difficulties, pureed diet |  |  |  |
| Indigestion/heartburn | 17 | 1 | 1 | 18 | | 1 | 0 | Medications noted: Ranitidine |  |  |  |
| Nausea/vomiting | 19 | 0 | 0 | 17 | | 2 | 0 | Today and after food, low appetite |  |  |  |
| Appetite/taste | 16 | 3 | 0 | 19 | | 0 | 0 | Very poor dietary supplements fortify diet, low appetite, appetite has decreased, puree diet |  |  |  |
| Bowel control | 15 | 4 | 0 | 18 | | 1 | 0 | Laxative/anti-motility agents noted as used include: lactulose, laxido, Senna, MGOH |  | 1 | smaller portions |
| Constipation | 15 | 4 | 0 | 15 | | 3 | 1 | Sometimes, laxatives needed, laxido used, can go up to 6 days with no bowels open |  | 1 | further checks |
| Sore throat | 19 | 0 | 0 | 19 | | 0 | 0 | Normal temp but looks hot |  | 0 |  |
| Respiratory problems | 18 | 1 | 0 | 17 | | 2 | 0 | Short of breath sometimes, anxiety |  |  |  |
| Optician visit in last year | 1 | 18 | 0 | 0 | | 19 | 0 | Dates provided for some residents last visit to opticians |  | NA |  |
| Dental visit in last year | 2 | 17 | 0 | 0 | | 18 | 1 | Dates provided for some residents last visit to dentists |  |  |  |
| Smoking | 19 | 0 | 0 | 19 | | 0 | 0 | None |  |  |  |
| Changes in smoking | 17 | 0 | 2 | 17 | | 0 | 2 | None |  |  |  |
| Drinking 6-8 cups/ day | 5 | 14 | 0 | 3 | | 16 | 0 | Lots of encouragement needed, staff push fluids onto daily, drinks well, very low fluid most days, 6 cups most days, good fluid intake |  |  |  |
| Snacking | 8 | 10 | 1 | 10 | | 8 | 1 | 1 date provided for latest diet diary, diet diary every day, obese, on diet control, only scheduled snacks, do not keep a diary  Weight reported as normal for 6 residents and not normal for 1 |  |  |  |
| Drinks sugar free | 7 | 11 | 1 | 9 | | 10 | 0 | Weight reported as normal for 6 residents and not normal for 2 |  |  |  |
| Intake: misses meals or leaves unfinished more than once / day | 15 | 4 | 0 | 18 | | 1 | 0 | 2 diet diary dates noted, weight subsequently indicated as normal for 3 residents and not normal for 1 |  |  |  |
| 2 or more meals eaten daily | 0 | 19 | 0 | 0 | | 18 | 1 | 1 diet diary date noted, weight subsequently indicated as normal for 1 resident |  |  |  |
| Fruit/veg intake daily | 5 | 14 | 0 | 6 | | 13 | 0 | Has fruit or veg daily, not daily, sometimes, offered but small intake |  |  |  |
| Milk intake: at least 1 pint per day. | 4 | 14 | 1 | 3 | | 16 | 0 | Milkshakes teas and supplements, enjoys coffee and milkshakes, takes very little prefers to drink water, soya, approx. 1 pint |  | 2 | encouraged to eat, forgets he has eaten |
| Vitamin D deficiency risk e.g. no sunlight exposure | 13 | 5 | 1 | 9 | | 10 | 0 | Don’t eat fish, takes invita d3 daily, veggie, takes supplements |  | 0 |  |
| Sun screen available | 9 | 9 | 1 | 6 | | 12 | 1 | Not outside often, does not go outside due to behaviour |  | 1 | waiting to see optician |
| Glasses for bright sunlight | 13 | 6 | 0 | 6 | | 13 | 0 | Sun hats available but not glasses, would refer/arrange if needed |  | 0 |  |
| Immunisations up to date | 0 | 16 | 3 | 0 | | 18 | 1 | 9 residents noted as having up to date influenza immunisation |  |  |  |
| Tablets crushed | 17 | 2 | 0 | 19 | | 0 | 0 | Crushed/mixed with food, liquid medication, compliant when not agitated |  | 1 | think of errors |
| Medicines administered regularly | 2 | 17 | 0 | 1 | | 18 | 0 | Some days so sleepy that misses some doses |  |  |  |
| Doses missed | 17 | 1 | 1 | 19 | | 0 | 0 | None |  | 0 |  |
| Non-prescription medicines used | 19 | 0 | 0 | 13 | | 0 | 6 | None |  | 0 |  |
| Recreational drug use | 19 | 0 | 0 | 19 | | 0 | 0 | None |  | 0 |  |
| Alcohol use | 18 | 0 | 1 | 19 | | 0 | 0 | One, very occasional, one or two lager in a week, 2 cans lager |  | 0 |  |
| Any other problems | 15 | 2 | 2 | 18 | | 1 | 0 | Being unable to do things independently due to Parkinsons, lack mental capacity, gout, posture, leg ulcers, unresponsive episodes (unknown cause, rarely occur) |  | 0 |  |
| Health problems important to you |  |  |  |  | |  |  | Eyesight gets upset and will say I can’t see, tracheotomy as unable to go out more, Communication, gets frustrated as unable to express to staff exactly what he wants, to be happy, being blind, being immobile and unable to visit places he used to, MS, speech, confusion, wishes to lose weight to help with mobility |  | 0 |  |
| Message for prescriber | 15 | 0 | 3 | 16 | | 0 | 3 | 6 residents noted as being unable to communicate/ unable to express self |  | 0 |  |

*Other actions were added to care plans or narrated to researchers in the debrief interviews.
**Data missing from last profiles has been substituted with available responses from 2nd profiles.

## Table D. Medicines and reported problems in first and last profiles.

This table illustrates the 4 themes and outcomes with cases and quotations.

Green – meds stopped or problem gone Red – new problem or medicine started

Possible causes of problems were derived by matching the problems listed on the Are-p profile with the ‘undesirable effects’ listed in the manufacturers’ literature or the British National Formulary, as summarised in ADRe-p’s supporting information.

Some preparations were noted as not administered on the MAR charts. All temperatures are *per axilla.*

| **Theme 1: clinical gain** | | | | | | |
| --- | --- | --- | --- | --- | --- | --- |
| The nurse said: | | | “It’s (ADRe-p) prompted us to go through this because of the ADRe-p, and then they worked with us on obviously identifying what the rash could be and giving the correct medication to address the rash. (…) The ADRe-p study has helped us identify little bits to push forward.” | | | |
| Case summary | | | This lady was very poorly, but by the end of the study was free of pain, seizures and the rash. She was administered antiepileptic drugs (AEDs) simultaneously with bulk laxatives; when diarrhoea was noted on ADRe-p, seizures ceased, presumably because the AED was then fully absorbed. | | | |
| **ID** | **Problems first ADRe-p** | **Possible causes** | **Medicines first ADRe-p Profile** | **Problems last ADRe-p** | **Possible causes** | **Medicines final ADRe-p Profile** |
| 1.3 | Temp 36^o^ | Incomplete contact | - 3m Cavilon™ Durable Barrier Cream, not given - Aymes® Shake Powder, 57g 2x a day with soya milk - Biotene® Oralbalance saliva replacement gel, not given apply as required - Cyclizine solution for injection ampoules, use 50mg every 8 hours - Diamorphine powder for solution for injection ampoules, use 2.5-5mg every 2 hours - Epaderm® ointment, not given apply as directed or use as soap substitute - Folic acid, take 5mg a day - Hydromol® ointment, not given apply to skin or use as soap substitute - Hyoscine hydrobromide, use 400mcg every 4 hours as required - Lamotrigine dispersible tablets, take 100mg 2x a day - Lamotrigine dispersible tablets, take 25mg 2x a day split tablet - Lorazepam, 1mg take half 4x a day when required - Macrogol® (compound) oral powder sachets, take 13.8g 2x a day in 125ml of water - Memantine tablets, take 20mg at night - Micralax® Micro-enema, administer 5ml rectally - Midazolam solution for injection ampoules, use 2.5mg as required - Mirtazapine orodispersible tablets, take 45mg a day - Movicol-Plain® oral powder sachets, take 13.7mg 2x a day in 125ml of water - Nutilis powder, not given use as required - Paracetamol, 10-20ml 4x a day - Ranitidine oral solution, take 10ml 2x a day - Sodium fluoride 1.1% dental paste, 2x a day - Tizanidine 2mg tablets, take half in the morning and 1 at night **splitting tablets – manufacturers supply oral solution** - White soft paraffin 13.2%/ liquid paraffin light 10.5% cream, 2x a day as required | Temp 35.6^o^ |  | - 3m Cavilon™ Durable Barrier Cream, not given - Aymes® Shake Powder, 57g 2x a day with soya milk - Biotene® Oralbalance saliva replacement gel, not given apply to gums & tongue as required - Cyclizine solution for injection ampoules, 50mg every 8 hrs as required - Diamorphine powder solution for injection ampoules, 2.5-5mg every 2 hrs as required - Epaderm® ointment, not given apply to skin or use as soap substitute - Folic acid, take 5mg once a day - Hydromol® ointment, not given apply to skin or use as soap substitute - Hyoscine hydrobromide solution, use 400mcg every 4 hrs as required - Lamotrigine 100mg tablets, take 1 2x a day - Lamotrigine 25mg tablets, take 1 2x a day - Lorazepam 1mg tablets, take half 4x a day as required – not used - Memantine, take 20mg once at night - Micralax® Micro-enema, 5ml administer once rectally - Midazolam solution for injection ampoules, use 2.5mg as required - Mirtazapine orodispersible tablets, take 45mg once a day - Movicol-Plain® oral powder sachets, take 13.7g 2x a day with 125ml of water - Nutilis powder, not given - Paracetamol, 10-20ml 4x a day - Piriton™ syrup, 5mls every 4-6 hours - Ranitidine oral solution, take 10ml 2x a day - Sodium fluoride 1.1% dental paste, 2x a day - Tizanidine 2mg tablets, take half in the morning & 1 at night - White soft paraffin 13.2%/ liquid paraffin light 10.5% cream, 2x a day as required |
| F | Unable to stand | Illness, diamorphine |  | Abnormal posture | Illness, lamotrigine, lorazepam, memantine |  |
| 65 | Poor balance/ co-ordination & unable to assist self | Diamorphine, lamotrigine, lorazepam, memantine |  | Unable to walk | Illness, diamorphine, lamotrigine, lorazepam, memantine |  |
|  | Rash | Diamorphine? – histamine release. Noted as allergic to opioids. Recognised and treated with Piriton™ |  | Poor balance/ co-ordination | Diamorphine, lamotrigine, lorazepam, memantine |  |
|  | Unable to mobilise | Illness, poor balance |  | Very hot to touch | Anticholinergic burden increased by Piriton™. |  |
|  | Epileptic convulsions | AEDs co-administered with bulk laxative  memantine, antidepressant |  | Sweating | Night sweats? This resident is very poorly. |  |
|  | Behaviour problems | Hyoscine, lamotrigine, lorazepam, memantine? |  | Behaviour problems (very vocal) | Hyoscine, lamotrigine, lorazepam, memantine? |  |
|  | Aggression | Hyoscine, lamotrigine, mirtazapine, lorazepam (rare), memantine? |  | Agitation and anxiety | Hyoscine, lamotrigine, lorazepam, memantine? |  |
|  | Agitation and anxiety | Hyoscine, lamotrigine, mirtazapine lorazepam, memantine? |  | Mood fluctuations | Hyoscine, lamotrigine, lorazepam, memantine? |  |
|  | Confusion | Hyoscine, cyclizine, lamotrigine, diamorphine, ranitidine, mirtazapine lorazepam, memantine? |  | Pain | Still in pain |  |
|  | Low energy | Illness, dehydration, diamorphine, mirtazapine, lamotrigine, |  | Eyesight problems | Illness |  |
|  | Pain | Opioid-induced hyperalgesia? |  | Swallowing difficulties | Dehydration, hyoscine, diamorphine, lamotrigine, |  |
|  | Blind in left eye | Illness |  |  |  |  |
|  | Doubly incontinent | Macrogol, antidepressant |  |  |  |  |
|  | Painful teeth and gums | Xerostomia?  Hyoscine, mental health meds, dehydration |  |  |  |  |
|  | Swallowing difficulties | Dehydration, hyoscine, diamorphine, lamotrigine, |  |  |  |  |
|  | Constipation | Hyoscine, diamorphine, (others less likely) |  |  |  |  |
|  | Diarrhoea | 1 laxative de-prescribed |  |  |  |  |
|  | Inadequate vitamin D intake |  |  |  |  |  |
|  |  |  |  |  |  |  |
|  | Pharmacist’s recommendations: none received | | | | | |
|  | Authors’ suggestions for discussion:   1. The patient remains in pain. How effective is diamorphine? Is it exacerbating the pain? Causing the rash? There is no evidence for assessment following administration, and this could be usefully charted. 2. Memantine may be contributing to several problems (e.g. lack of ordination, behaviour problems): is there still a therapeutic rationale? 3. The high anticholinergic burden, particularly hyoscine (not recommended for PWD), may be responsible for the behaviour problems and distress, and possibly the high peripheral temperature. 4. Splitting tablets may mean little is received: liquid formulations would ensure the prescribed dose is received. | | | | | |
| **Theme 2: Prescribing changes** | | | | | | |
| The nurses said | | | “it [ADRe-p] helps to know their [residents’] concerns (…) to know them more.” | | | |
| Case summary | | | This lady had been confined to a wheel chair since contracting Guillain-Barré syndrome, and had complex needs. She was mentally alert and aware of her situation. Documentation of problems on ADRe-p triggered deprescribing of iron, reduced dose of anti-coagulants, increased prescribing to achieve asthma control, catheter care improved, rash and pain. | | | |
| **ID** | **Problems first ADRe-p** | **Possible causes** | **Medicines first ADRe-p Profile** | **Problems last ADRe-p** | **Possible causes** | **Medicines final ADRe-p Profile** |
| 2.2 | BP 144/74 | Salbutamol (diastolic not raised), pregabalin | - Adcal® D3 Dissolve eff tab, take 1 2x a day - Carbocisteine, take 375mg once a day - Citalopram, take 40mg once a day - Ferrous Fumarate, take 210mg once a day - Lactulose, take 10ml 2x a day - Lansoprazol, take 30mg once a day - Letrozole, take 2.5mg once a day - Optiflo® G sterile soln, take 2 via catheter 2x a week - Paracetamol, 500mg take 2 up to 4x a day when required, 2 given. - Pregabalin 200mg caps, take 2 a day - Pregabalin 50mg caps, take 1 at night - Ramipril 5mg caps, take 1 2x a day - Rivaroxaban, take 20mg once a day - Salbutamol, 2.5mg 4x a day neb, when required. 1 use seen. | BP 140/73 | Salbutamol (diastolic not raised), pregabalin | - Adcal® D3 dissolve eff tab, take 1 2x a day - Carbocisteine, take 375mg once a day - Citalopram, take 40mg once a day - ~~Ferrous Fumarate, take 210mg once a day~~ marked as course completed but still on chart, crossed off. - Lactulose, take 10ml 2x a day - Lansoprazol, take 30mg once a day - Laxido® Orange oral powder, take 1 2x a day - Letrozole, take 2.5mg once a day - Optiflo® G sterile soln, 2 bottles 2x a week - Optilube™ Active Sterile gel, not given - Paracetamol, 500mg take 2 up to 4x a day when required, 1 given - Pregabalin 200mg caps, take 2 a day - Pregabalin 50mg caps, take 1 at night - Ramipril 5mg caps, take 1 2x a day - Rivaroxaban, take 10mg once a day DOSE REDUCED - Salbutamol, 2.5mg 4x a day when required, none given - Ventolin™ Evohaler, take 1-2 puffs, 4x a day as required |
| F | BMI 31.2 | Pregabalin, citalopram |  | BMI 31.6 | Pregabalin, citalopram |  |
| 71 | Unable to stand | Illness |  | Unable to stand | Illness |  |
|  | Hand tremors affecting activities of daily living | Salbutamol,  citalopram, high dose |  | Hand Tremors affecting activities of daily living | Salbutamol,  Citalopram, high dose |  |
|  | Unable to walk | Illness |  | Immobile | Illness |  |
|  | Feels cold all the time | Vasodilated, infection, pregabalin, hypoxia |  | Feels cold | Vasodilated, infection, pregabalin |  |
|  | Rash & swelling (cellulitis) | Oedema and pregabalin, rivaroxaban, hypoxia |  | Anxiety for the future | Genuine distress, need for additional psychosocial support.  Pregabalin, oedema, infection, cancer therapies, salbutamol |  |
|  | Anxiety (worries about dying) | Genuine distress, need for additional psychosocial support.  Pregabalin, oedema, infection, cancer therapies, salbutamol, hypoxia |  | Mood fluctuations | Pregabalin, citalopram, salbutamol |  |
|  | Mood fluctuations | Pregabalin, citalopram, salbutamol, hypoxia |  | Vision problems (wears glasses) | Pregabalin can cause eye problems. |  |
|  | Low energy & weakness when feeling sad | Rivaroxaban, pregabalin, citalopram, cancer therapies, hypoxia |  | Suprapubic catheter |  |  |
|  | Pain | Pregabalin |  | CA Breast |  |  |
|  | Vision problems (wears glasses) | Pregabalin can cause eye problems. Letrozole can cause cataract. |  | Not eating fruit & veg everyday |  |  |
|  | Urination problems (suprapubic catheter) | Illness. The problem is blockage, likely exacerbated by calcium tablets and dehydration. |  | Inadequate Vitamin D intake | Supplemented, so not a problem |  |
|  | Guillain-Barré syndrome |  |  | Asthma |  |  |
|  | CA Breast |  |  |  |  |  |
|  | Short of Breath (asthmatic) | Under treatment, citalopram, anaemia |  |  |  |  |
|  | Not eating fruit & veg everyday | Loss of appetite, but this is not noted |  |  |  |  |
|  | Dehydration risk | Ramipril |  |  |  |  |
|  | Pharmacist’s recommendations: none received | | | | | |
|  | Authors’ suggestions for discussion:   1. The suprapubic catheter has been blocking, yet a prescription containing calcium is continued. Vitamin D is needed, but preparations without calcium are more likely to be beneficial. 2. Anaemia – please consider possible role of rivaroxaban (dose now reduced). Dose of oral iron was low. 3. The dose of pregabalin is high. Given the tracheostomy and suprapubic catheter, is there an alternative to a drug predisposing to infections, which are impacting on quality of life? 4. Tremor: please check if this follows salbutamol administration. 5. Lactulose administered, but no constipation. No other records of bowel movements found. 6. We did not locate a thyroid function check, despite weight gain, feeling cold, behaviour problems and tremor. 7. The mental health reports are concerning; it isn’t clear what counselling support has been offered or taken up. 8. A review of the tracheostomy and respiratory medicines would be timely. Oxygen saturations is low at times. | | | | | |
|  | | | | | | |
| **Theme 3: multidisciplinary working** | | | | | | |
| The nurses said: | | | “I’m just thinking, this medication, does she still need this? Does she present any symptoms for this medication?” | | | |
| Case summary | | | This lady was frail, confused and cognitively impaired. Her behaviour was challenging. Application of ADRe-p and its supporting information reduced her stiffness and pain. Multiple prescribers were involved (see text). The pharmacist was concerned over the risk of fractures. | | | |
| **ID** | **Problems first ADRe-p** | **Possible causes** | **Medicines first ADRe-p Profile** | **Problems last ADRe-p** | **Possible causes** | **Medicines final ADRe-p Profile** |

| 3.3 | BMI 31 | Quetiapine | - Alendronic acid 70mg tabs, take 1 weekly with water in the morning before eating - Co-Careldopa 12.5mg+50mg (carbidopa & levodopa), take 1 at 6pm - Daktacort® cream, apply 2x a day - Darifenacin 15mg tab, take 1 daily - Fenbid® 5% gel, apply 3x a day - Glycerol 4g, use rectally as required not used - Invita® D3 800 units, take 1 every morning - Laxido® Orange oral powder, take 1 sachet in water 2x a day (morning & teatime 5pm) - Medi Derma-S® Barrier Cream, not given - Paracetamol 5ml, take 2-4 up to 4x a day as required - Quetiapine 25mg tabs, take 1 at night - Rotigotine 2mg, apply patch every 24hrs - Senna 7.5mg tabs, take 1-2 at night when required - Sertraline 50mg tabs, take 1 at 8am & 1 midday - Sinemet Plus 25mg+100mg tabs, take 2 3x a day at 6am, 10am and 2pm - Sodium fluoride 5000ppm toothpaste, use 2x a day | BP 108/62, but 112/76 by end of study. | Dopamine agonists – worsened with increased dose, dehydration | - Alendronic acid 70mg tabs, take 1 weekly with water in the morning before eating - Co-Careldopa (carbidopa+L-dopa) 12.5mg+50mg tabs, take 1 at 6pm - Darifenacin 15mg tab, take 1 daily - Fenbid 5% gel, apply 3x a day - Glycerol 4g, use rectally as required - Half Sinemet Cr 25mg+100mg tabs, take 1 at 10pm - Invita® D3 800 units, take 1 every morning - Laxido® Orange oral powder, take 1 sachet in water 2x a day (morning & teatime 5pm) - Medi Derma-S® Barrier Cream, not given - Paracetamol 5ml, take 2-4 up to 4x a day as required - Quetiapine 25mg tabs, take 1 at night - Rotigotine 2mg, apply 1 patch daily - Senna 7.5mg tabs, take 1-2 at night when required - Sertraline 100mg tabs, take 1 each day DOSE NO LONGER DIVIDED by study end increased to 150mg *mane* for agitation - Simvastatin 20mg tabs, take 1 every evening - Sinemet Plus 25mg+100mg tabs, take 1 at 6pm - **Co-careldopa 12.5mg+50mg tabs, 1 at 6pm added by end of study** - **Half sinemet cr 25mg+100mg tabs, take 1 at 10pm added after last profile** - Sinemet plus 25mg+100mg tabs, take 2 3x a day at 6am, 10am and 2pm - Sodium fluoride 5000ppm toothpaste, use 2x a day |
| --- | --- | --- | --- | --- | --- | --- |
| F | Oxygen saturation 95% | Cardiovascular or respiratory condition |  | BMI 31.2, and 30.8 by end of study | Quetiapine, |  |
| 81 | Hand tremors (Parkinson’s) |  |  | Girth 120cm | Quetiapine |  |
|  | Abnormal movements at rest | Quetiapine |  | Oxygen saturation 93%, 94% by end of study | Hypotension |  |
|  | Abnormal gait | Parkinson’s, sertraline |  | Hand tremors | Parkinson’s |  |
|  | Poor balance/ co-ordination affecting daily living activities | Parkinson’s, quetiapine |  | Feet shuffling | Parkinson’s, quetiapine |  |
|  | Behaviour problems | Akathisia? / quetiapine,  dopamine agonists, sertraline, hypoxia |  | Abnormal movements at rest | Quetiapine |  |
|  | Anxiety | Dopamine agonists, sertraline, quetiapine, hypoxia |  | Abnormal posture & gait | Parkinson’s, sertraline |  |
|  | Confusion | Antimuscarinic (darifenacin), dopamine agonists,  quetiapine, sertraline, hypoxia, dehydration |  | Poor balance/co-ordination affective daily living activities | Parkinson’s, quetiapine |  |
|  | Mood fluctuations | Dopamine agonists, sertraline, quetiapine |  | Cognitive decline | Antimuscarinic (darifenacin), dopamine agonists,  quetiapine, sertraline, hypoxia, dehydration |  |
|  | Restless | Dopamine agonists, sertraline, quetiapine |  | Sweating | Sinemet, increased dose |  |
|  | Sleep problems (variable sleep pattern) | Dopamine agonist, sertraline, quetiapine |  | Behaviour problems | Akathisia? / quetiapine,  dopamine agonists, sertraline, hypoxia |  |
|  | Dizziness | Paracetamol, hypoxia, hypotension |  | Agitation/ anxiety | Dopamine agonists, sertraline, quetiapine, hypoxia |  |
|  | Falls | Dopamine agonist,  quetiapine, sertraline, hypotension, hypoxia, dizziness, confusion |  | Confusion | Antimuscarinic (darifenacin), dopamine agonists,  quetiapine, sertraline, hypoxia, dehydration |  |
|  | Pain | Possibly joint stiffness |  | Mood fluctuations | Increased sinemet  Dopamine agonists, sertraline, quetiapine |  |
|  | Vision problems (wears glasses) | Sertraline, antipsychotic, darifenacin |  | Hallucinations | Increased sinemet, darifenacin |  |
|  | Incontinence | Sertraline, antipsychotic, darifenacin, Parkinson’s |  | Dizziness | Hypotension, hypoxia, paracetamol |  |
|  | Dehydration risk | Tremor |  | Falls | Dopamine agonist,  quetiapine, sertraline, hypotension, hypoxia, dizziness, confusion |  |
|  | Doesn’t drink enough milk |  |  | Pain (shoulder) | Joint stiffness, Parkinson’s |  |
|  |  |  |  | Vision problems (glasses) | Sertraline, antipsychotic, darifenacin |  |
|  |  |  |  | Dehydration risk | Tremor |  |
|  |  |  |  | Doesn’t drink enough milk |  |  |
|  |  |  |  |  |  |  |

|  | Pharmacist’s recommendations:  No calcium supplement prescribed, speak to care home staff to see if mobile & how much calcium is taken in diet. Speak to GP to review on next visit to decide if calcium supplement is necessary. (No evidence this was done.) | | | | | | | | | |
| --- | --- | --- | --- | --- | --- | --- | --- | --- | --- | --- |
|  | Authors’ suggestions for discussion:   1. Follow pharmacist’s advice on calcium and vitamin D supplementation – dizziness and falls reported. 2. Dehydration and worsening hypotension indicate the need for an accurate record of fluid intake. 3. Hypoxia is worsening, and may be contributing to confusion and anxiety. Close review might be indicated. 4. Confusion likely worsened by darifenacin’s antimuscarinic properties. Incontinence appears controlled, but darifenacin may be prescribed for Parkinson’s: suggest review. 5. It is difficult to control long-standing Parkinson’s without adversely affecting mental health, and problems need to be kept under close review to facilitate achieving the balance. 6. The value of quetiapine to control behaviour should be jointly reviewed by the MDT: lorazepam PRN is likely to be the better option. Quetiapine is likely contributing to falls, dizziness, ataxia, confusion, anxiety, and restlessness. | | | | | | | | | |
|  |  | | | | | | | | | |
|  | **Theme 4 : costs and resources** | | | | | | | | | |
|  | Case summary: This lady was frail. The pharmacist intervened to deprescribe and prevent further falls. Discontinuation of buprenorphine likely ended seizures, further reducing the risk of falls. | | | | | | | | | |
|  | The nurses said: “I think it’s a useful tool, it gives us a much bigger and better idea of what we should be looking for apart from our normal observations (…) every student, I’d be happy to give them one of these to say this is what you need to be thinking about when you’re looking at medications.” | | | | | | | | | |
| **ID** | **Problems first ADRe-p** | **Possible causes** | **Medicines first ADRe-p Profile** | | | **Problems last ADRe-p** | **Possible causes** | **Medicines final ADRe-p Profile** | |  |
| 2.8 | HR 94, 68 by 3^rd^ profile | Possible heart failure, aripiprazole | | - Aripiprazole 5mg tabs, take half a tablet daily - Buprenorphine 5micrograms, apply 1 patch weekly 3 given - Duraphat® 5000 toothpaste, use 2x a day - Levetiracetam 250mg tabs, take 1 2x a day (morning & night) - Memantine 20mg tabs, take 1 daily - Mirtazapine 30mg tab, take 1 at night - Paracetamol 500mg sol tab, take 2 up to 4x a day as required - Pregabalin 100mg caps, take 1 2x a day (morning & night) - Senna 7.5mg tabs, take 2 at night - Sodium fluoride 5000ppm toothpaste, use 2x a day - Zopiclone 7.5mg tabs, take 1 at night when required 3 given | Feet shuffling | | Aripiprazole- tablet split when oral solution could be used: exacerbated by pregabalin, levetiracetam, mirtazapine, memantine | | - Aripiprazole 5mg tabs, take half a tablet daily - Levetiracetam 250mg tabs, take 1 2x a day (morning & night) - Memantine 20mg tabs, take 1 daily - Mirtazapine 30mg orodispersible tab, take 1 at night - Pregabalin 100mg caps, take 1 2x a day (morning & night) - Senna 7.5 mg tabs, take 2 at night - Sodium fluoride 5000ppm toothpaste, use 2x a day |  |
| F | BP 161/97 117/59 by 3^rd^ profile | Pregabalin?, memantine | |  | Abnormal posture | | Aripiprazole?, parkinsonism exacerbated by pregabalin, levetiracetam, mirtazapine & memantine | |  |  |
| 80 | Temp 36^o^ | In complete contact – resolved by 3^rd^ profile | |  | Poor balance/ co-ordination affecting daily living activities | | Aripiprazole? ,parkinsonism exacerbated by pregabalin, levetiracetam mirtazapine, memantine | |  |  |
|  | Feet shuffling | Aripiprazole- tablet split when oral solution could be used: exacerbated by pregabalin, levetiracetam, mirtazapine, buprenorphine & memantine | |  | Cognitive decline | | Antipsychotic, AEDs, opioid | |  |  |
|  | Abnormal posture | Aripiprazole? parkinsonism exacerbated by pregabalin, levetiracetam, mirtazapine & memantine | |  | Feeling the cold/ cold to touch | | Immobility due to gait problems, antipsychotic | |  |  |
|  | Abnormal gait (very small steps) | Aripiprazole?, parkinsonism exacerbated by pregabalin, levetiracetam mirtazapine, buprenorphine & memantine | |  | Swelling/ oedema (ankles) | | Aripiprazole?, HF (tachycardia), memantine, pregabalin | |  |  |
|  | Poor balance/ co-ordination affecting daily living activities | Aripiprazole?, parkinsonism exacerbated by pregabalin, levetiracetam mirtazapine, memantine, buprenorphine & zopiclone | |  | Anxiety/ nervousness | | Pregabalin, levetiracetam, mirtazapine | |  |  |
|  | Cognitive decline | Antipsychotic, AEDs, opioid | |  | Confusion | | Pregabalin, memantine, mild hypoxia | |  |  |
|  | Feeling the cold/ cold to touch | Immobility due to gait problems, antipsychotic, opioid | |  | Low energy/ weakness | | Pregabalin, levetiracetam, | |  |  |
|  | Swelling/ oedema (ankles) | Aripiprazole?, HF (tachycardia), memantine, pregabalin | |  | Hallucinations | | Memantine | |  |  |
|  | Convulsions | Memantine (rare – but see cautions), AEDs, antipsychotic, opioid | |  | Dizziness | | Memantine, levetiracetam, antipsychotic, pain | |  |  |
|  | Hallucinations | Memantine, opioids | |  | Falls | | Aripiprazole?, parkinsonism exacerbated by pregabalin, levetiracetam, mirtazapine, memantine | |  |  |
|  | Agitation/ anxiety | Pregabalin, levetiracetam, buprenorphine, mirtazapine | |  | Pain | | Joint stiffness due to parkinsonism | |  |  |
|  | Panic attacks | Hypoxia?, buprenorphine | |  | Incontinent | | Antipsychotic, mirtazapine | |  |  |
|  | Confusion | Pregabalin, memantine, zopiclone, opioid, mild hypoxia | |  | Short of breath | | HF? – ankle swelling above, 97% sats, memantine, mirtazapine, | |  |  |
|  | Low energy/ weakness | Pregabalin, levetiracetam, opioids | |  | Hyper salivation (due to posture) | | Aripiprazole | |  |  |
|  | Dizziness | Memantine, levetiracetam, antipsychotic, pain | |  | Constipation | | Aripiprazole, memantine | |  |  |
|  | Falls | Aripiprazole?, parkinsonism exacerbated by pregabalin, levetiracetam, mirtazapine, buprenorphine, zopiclone & memantine | |  | Doesn’t eat fruit & veg everyday | | Anorexia due to constipation | |  |  |
|  | Pain | Consider long-term opioid, joint stiffness due to Parkinsonism | |  | Doesn’t drink enough milk | |  | |  |  |
|  | Short of breath (anxiety) | HF? – ankle swelling above, 97% sats, mirtazapine, memantine, buprenorphine | |  | Inadequate vitamin D intake | |  |  |  |  |
|  | Hyper salivation | Aripiprazole | |  |  | |  |  |  |  |
|  | Constipation | Opioid, aripiprazole, memantine | |  |  |  |  |  |  |  |
|  | Doesn’t eat fruit & veg everyday | Anorexia due to constipation or opioid | |  |  |  |  |  |  |  |
|  | Doesn’t drink enough milk | Anorexia due to constipation or opioid | |  |  |  |  |  |  |  |
|  | Inadequate vitamin D intake |  | |  |  |  |  |  |  |  |
|  | Pharmacist’s recommendations:  Constipation, common ADR of Butrans™, patient has Senna every night, GP to review laxatives if home feel constipation is problematic.  Dizziness and falls could be due to more than one of prescribed meds, all to be reviewed by GP if problematic. Information passed to GP after last PADRe.  Hyper salivation common side effect of aripiprazole, care home staff to request review from GP if problematic  BP on low side, patient does not take any antihypertensives  Patient seems to drink plenty, care home staff to ensure this is the case | | | | | | | | | |
|  | Authors’ suggestions for discussion:  As indicated by the pharmacist,   1. Urgent med review needed, to reduce posture & movement problems and dizziness before the resident falls again (aripiprazole, pregabalin, levetiracetam, mirtazapine, memantine). 2. PADRe identified signs of heart failure. The need for memantine, the antipsychotic and mirtazepine should be considered. 3. Pain persists: further description is needed. 4. Hypertension is no longer present, but monitoring should be continued.   Tablet splitting leads to uneven dosing of aripiprazole: a liquid is available. | | | | | | | | | |
|  |  | | | | | | | | | |
