# Supplementary material for: Nobody ever questions—Polypharmacy in care homes: A mixed methods evaluation of a multidisciplinary medicines optimisation initiative
Source: PLoS One. 2021 Jan 7;16(1):e0244519. doi: 10.1371/journal.pone.0244519 (PMC7790299; doi:10.1371/journal.pone.0244519)
Supplement: S3 File — (DOCX) [file pone.0244519.s003.docx]

S3 File: Interview guides

These questions are guides only SJ 29.10.2018

## Introductory Interview with Nurses

Experience

1. Have you had any formal training in looking out for adverse drug reactions (ADRs) and side effects of medicines or multiple medicines?
2. Can you think of any patients that may have had or be experiencing adverse drug reactions or adverse side effects from their medicines?

Prompts: patients that are too sleepy / over-sedated, having falls, feeling sick, complaining of a headache etc.

1. If so, what action did you take if you thought something was not quite right?

*Prompts: eg let the GP know, discuss with colleagues, make a note in the care plan?*

1. What was the outcome of taking these actions? Did this help at all?

Learning

1. Following initial discussions regarding the ADRe-p Study, what do you think about it? *Prompts: Do you think it will help the patients? Was there anything that particularly motivated you to participate in the study?*
2. Do you have any worries about participating in the study? Can you think of any possible challenges?
3. Do you feel ready to start the study and use the ADRe Profile? If yes, why? If not, why not?

Process (Action/Behaviour)

1. Have you or the other staff made changes to prepare for the study?

*Prompts: discussed it in team meetings or hand overs?*

Outcomes/Improvements

1. Apart from possibly finding ADRs/side effects of polypharmacy in residents, do you think anything else will change from participating in the study?

## Debriefing / end of study Interview Guide with nurses, pharmacists

Experience

1. In general, how are you finding being part of the ADRe-p Study?

*Prompts: eg time, working with the ADRe team, pharmacists, GPs, being with the patients and consultees.*

1. Did ADRe help you assess the residents and identify possible side effects?
2. Were there any challenges?

*Prompts: time pressures/interruptions/distractions/equipment e.g. sphygs/completing over several sessions.*

1. How often have you used the ADRe-p Profile to date?
2. How was using it? (For you/for the residents?). Do you have any suggestions on how we can improve the Profile or supporting information?
3. What about the other work?

*Prompts: consenting the patients, contacting GPs*

1. Looking back on the training at the beginning: How could we have improved it to make it easier for you to use the Profile/be part of the study?

Learning

1. What helped you while you were in the ADRe-p Study?

Prompts: The teaching at the start, the researchers, the team as a whole, support from the group, the benefits to residents?

Process (Action/Behaviour)

1. Did you make any changes/actions to care following the use of the Profile/being part of the Study?

*Prompts: to how many people?/Did ADRe-p prompt you to contact anyone? Dentists / opticians / doctors / pharmacists? Were changes made in care plans after ADRe?*

Outcomes/Improvements

1. Do you think that the tool led to clinical changes? clinical outcomes or improvements? This could be for the patients, for the nursing team etc.
2. Has ADRe changed your thinking / focus on medicines?

If so, could you give me an example?

1. Has ADRe affected communication with pharmacists and prescribers?

If so, could you give me an example?

1. Does ADRe stimulate actions?

If so, could you give me an example?

1. Would you like the tool to continue to be used in the care home?

## Service Users

1. What made you want to take part in this study?
2. *Have you had any experience of any adverse effects of medicines?*

*Please describe*

1. If so, what action did you take if you thought something was not quite right?

*Prompts: eg let the GP or nurses know?*

1. What was the outcome of taking these actions? Did this help at all?
2. What has been your experience of this study?
3. Do you think that ADRe led to clinical changes? clinical outcomes or improvements? This could be for the patients, for the nursing team etc.

If so, could you give me an example?
