# Supplementary material for: Nobody ever questions—Polypharmacy in care homes: A mixed methods evaluation of a multidisciplinary medicines optimisation initiative
Source: PLoS One. 2021 Jan 7;16(1):e0244519. doi: 10.1371/journal.pone.0244519 (PMC7790299; doi:10.1371/journal.pone.0244519)
Supplement: Attachment — Submitted filename: Response to reviewers.docx [file pone.0244519.s004.docx]

# Response to reviewers

# Nobody ever questions – polypharmacy in care homes: a mixed methods evaluation of a multidisciplinary medicines optimisation initiative

Short title: The adverse drug reaction profile for polypharmacy

Rebuttal letter / response to reviewers [PONE-D-20-32601] - [EMID:148862874e6b707a]

24.11.20

Dear Professor Vaismoradi,

Thank you for the return of our manuscript. We are very grateful for the reviewers’ support and suggestions. We have done our best to comply, as tabulated below.

We feel that clinicians need to be aware of the importance of monitoring patients for any adverse effects of prescribed medicines. Publication in *PlosOne* would stimulate prescribers to take a proactive approach to medication review for people in long-term care.

We hope that readers will concur with the reviewers:

The work has not been submitted elsewhere.

Thank you for your time spent on this paper.

Yours,

Sue Jordan, on behalf of all authors

Changes Made

We have tracked in the text where we have made the suggested changes. The paper has been submitted with and without tracks.

**Journal Requirements:**

1.) Please ensure that your manuscript meets PLOS ONE's style requirements, including those for file naming. The PLOS ONE style templates can be found at

<https://journals.plos.org/plosone/s/file?id=wjVg/PLOSOne_formatting_sample_main_body.pdf> and

<https://journals.plos.org/plosone/s/file?id=ba62/PLOSOne_formatting_sample_title_authors_affiliations.pdf>

*We have done our best to comply. We are unaware of any outstanding issues, but are happy to modify if required.*

2.) Please include additional information regarding the interview guide or script used in the study and ensure that you have provided sufficient details that others could replicate the analyses. For instance, if you developed a guide as part of this study and it is not under a copyright more restrictive than CC-BY, please include a copy, in both the original language and English, as Supporting Information.

*This has been appended as supplementary material, S3 file.*

3.)We note that you have indicated that data from this study are available upon request. PLOS only allows data to be available upon request if there are legal or ethical restrictions on sharing data publicly. For information on unacceptable data access restrictions, please see <http://journals.plos.org/plosone/s/data-availability#loc-unacceptable-data-access-restrictions>.

 In your revised cover letter, please address the following prompts:

a) If there are ethical or legal restrictions on sharing a de-identified data set, please explain them in detail (e.g., data contain potentially identifying or sensitive patient information) and who has imposed them (e.g., an ethics committee). Please also provide contact information for a data access committee, ethics committee, or other institutional body to which data requests may be sent.

The Data Availability Statement now reads:

Data are provided within the supplementary tables. Ethical restrictions have been imposed on data sharing by the NHS Research Ethics Committee that approved this study. The data contain potentially identifying and sensitive information. The data used in this study are available to the research data community at <https://zenodo.org/record/4090384#.X4hKptZFzLZ> Swansea University, Swansea, UK.

All proposals to view the data are subject to review by Swansea University’s Research Governance department and the PI. Before any data can be accessed, approval must be given.

The application process is *via* the Academic Lead for Research Integrity Research Engagement & Innovation Services, Swansea University and the PI or Neil Carter.

Contacts: Swansea University, Swansea SA2 8PP

- Tel: +44 /0 1792 606060 and 518541 or 295610
- Email: [researchgovernance@swansea.ac.uk](mailto:researchgovernance@swansea.ac.uk), [s.e.jordan@swansea.ac.uk](mailto:s.e.jordan@swansea.ac.uk) or [n.carter@swansea.ac.uk](mailto:n.carter@swansea.ac.uk)

The research instrument used in the study is available for clinical use without charge *via* the project website: <http://www.swansea.ac.uk/adre/>

b) If there are no restrictions, please upload the minimal anonymized data set necessary to replicate your study findings as either Supporting Information files or to a stable, public repository and provide us with the relevant URLs, DOIs, or accession numbers. Please see <http://www.bmj.com/content/340/bmj.c181.long> for guidelines on how to de-identify and prepare clinical data for publication. For a list of acceptable repositories, please see <http://journals.plos.org/plosone/s/data-availability#loc-recommended-repositories>.

*Our data are restricted, as above.*

Response to reviewers

We are very grateful for the detailed consideration given to our paper, and have responded to the points raised below.

| Reviewer #1: |  |
| --- | --- |
| Dear authors,  I would like to thank you for giving me the opportunity to review this high-quality manuscript. This work is well designed and well written manuscript that improve patient’s safety in home care using the involve of nurses in the Adverse Drug Reaction Profile for polypharmacy. I have some few comments: | Thank you for your support. |
| In introduction- can you give more literature about using of ADRe-p in health care professions particularly in nurses. | We have moved and added some text on this to the introduction, last sentence:  Our intervention, the ADRe Profile [2-4], achieves this by uniting the tacit, experiential knowledge of nurses and carers with records of the patients’ clinical problems and possible causes, in a form that can be shared within the multidisciplinary team [2-7] (Fig 1).  This entails moving Fig 1. |
| In methods- line 125: please enter the full name of the abbreviation. | Standards for Quality Improvement Reporting Excellence – done |
| Line 142: Is there range for polypharmacy?? In the introduction you report a statistic about polypharmacy in which > 4 was used for polypharmacy. Why you used >3 for polypharmacy? please provide more clarification. | Definitions differ. We explain in the inclusion criteria:  This definition of polypharmacy is used by Cochrane reviewers [27]. We were unable to adopt definitions based on appropriateness or duration of prescriptions before gaining consent to view records. |
| What is difference between Line 142 as a inclusion criteria and line 147 as a exclusion criteria?? In my opinion both of them are same. Please use only a definite inclusion criterion for this item. | The line is deleted – thank you. |
| In results- Figure 2a and 2b: arrows are crooked. | The figures have been redrawn and new tif files uploaded. |
| Reviewer #2: Thank you for the opportunity to review this study. The structure of the manuscript is well put together and it is written up clearly. The only challenge to the manuscript is that it needs a bit of revising for punctuation, inclusion criteria, and discussion section. | Thank you |
| - Please, pay attention to punctuation. For instance in lines 268,284 and so on. | Line 268, now 269, ‘and’ has been substituted for ‘or’.  Line 284 – no errors identified.  Line 455, now 468 – full stop substituted for semicolon  Line 458, now 459 – definite article removed  Line 460, now 464 – ‘however’ substituted for ‘but’  Arabic numerals replaced with text throughout. |
| - Inclusion criteria: state rationale for why you chose >3 prescribed medicines daily. | We added this statement, as above:  This definition of polypharmacy is used by Cochrane reviewers [27]. We were unable to adopt definitions based on appropriateness or duration of prescriptions before gaining consent to view records. |
| - Please, at the first paragraph, describe the aim of the study. | We have added to start of discussion:  We aimed to identify changes following nurses’, carers’ and pharmacists’ use of ADRe-p. |
